# Endemic, Notifiable Bioterrorism-Related Diseases, United States, 1992–1999

**DOI:** 10.3201/eid0905.020477

**Published:** 2003-05

**Authors:** Man-huei Chang, M. Kathleen Glynn, Samuel L. Groseclose

**Affiliations:** * Centers for Disease Control and Prevention, Atlanta, Georgia, USA

**Keywords:** bioterrorism, incidence, research

## Abstract

Little information is available in the United States regarding the incidence and distribution of diseases caused by critical microbiologic agents with the potential for use in acts of terrorism. We describe disease-specific, demographic, geographic, and seasonal distribution of selected bioterrorism-related conditions (anthrax, botulism, brucellosis, cholera, plague, tularemia, and viral encephalitides) reported to the National Notifiable Diseases Surveillance System in 1992–1999. Tularemia and brucellosis were the most frequently reported diseases. Anthrax, plague, western equine encephalitis, and eastern equine encephalitis were rare. Higher incidence rates for cholera and plague were noted in the western United States and for tularemia in the central United States. Overall, the incidence of conditions caused by these critical agents in the United States is low. Individual case reports should be considered sentinel events. For potential bioterrorism-related conditions that are endemic and have low incidence, the use of nontraditional surveillance methods and complementary data sources may enhance our ability to rapidly detect changes in disease incidence.

In 2001, anthrax cases associated with the intentional distribution of *Bacillus anthracis* spores through the postal system re-emphasized that the deliberate exposure of humans to biologic agents can happen in the United States (*1*,*2*). Before the 2001 bioterrorism-associated anthrax events, terrorist attacks (e.g., the bombings of the World Trade Center in New York City in 1993, the Federal Building in Oklahoma City in 1995, and the Olympic Games in Atlanta in 1996; and an increase in intentional anthrax exposure hoaxes [*3*]) had already created substantial media and public attention because they highlighted our susceptibility to domestic terrorism, including bioterrorism. In addition, smaller focused acts of bacteriologic criminal assault had occurred in the United States, including the intentional contamination of salad bars with *Salmonella* organisms in 1984 in Oregon (*4*) and of muffins and pastries with *Shigella* organisms in Texas in 1996 (*5*); these acts served as a wake-up call announcing the threat of domestic bioterrorism. All of these events led the United States to revisit and update a national plan for bioterrorism preparedness and response in the late 1990s. In defining the role of the public health community in the detection of and response to bioterrorism, the Centers for Disease Control and Prevention (CDC) identified 10 major areas of need. One of these areas is ensuring reliable and timely disease surveillance and reporting to detect and investigate outbreaks (*6*).

In response to global bioterrorism threats, CDC has proposed a list of critical biologic agents that have potential for use in a terrorist incident (*6*–*9*). This list includes a wide range of biologic agents and prioritizes pathogens into three categories on the basis of their potential to affect the public’s health, their potential for dissemination, and special needs for effective public health intervention. Prioritization of bioterrorism “threat” agents facilitates coordinated planning efforts for preparedness and response to bioterrorism at the local, state, and federal levels.

Using this guidance, public health systems can address the threat of bioterrorism by increasing healthcare sector awareness of and surveillance for these bioterrorism-related agents and the diseases they cause (*10*). In the United States, public health surveillance for conditions caused by the identified critical biologic agents is conducted in multiple ways. Although data regarding these agents are reported to different national surveillance systems at CDC, no single system is specifically designed for conducting surveillance for all bioterrorism-related agents or conditions. However, many states have routinely conducted surveillance for some of these conditions and report incidence data to CDC’s National Notifiable Diseases Surveillance System (NNDSS) each week (Table 1).

**Table 1:** Number of reported cases and number of states reporting conditions caused by critical biological agents, National Notifiable Diseases Surveillance System, United States, 1992–1999^a,b^

**Y**	**Anthrax**	**Botulism, foodborne**	**Botulism, Other^c^**	**Brucellosis**	**Cholera**	**Encephalitis, eastern equine**	**Encephalitis, western equine**	**Plague**	**Tularemia**
**1992–1999**									
**Total cases**	**1**	**223**	**148**	**813**	**223**	**29**	**1**	**77**	**885**
**1992**									
**Cases**	**1**	**21**	**4**	**105**	**103**	**N**	**N**	**13**	**159**
**States reporting**	**1**	**8**	**2**	**22**	**12**	**N**	**N**	**7**	**26**
**States requiring reporting**	**52**	**52**	**52**	**52**	**52**	**N**	**N**	**52**	**52**
**1993**									
**Cases**	**0**	**27**	**5**	**120**	**25**	**N**	**N**	**11**	**132**
**States reporting**	**0**	**10**	**3**	**23**	**11**	**N**	**N**	**4**	**24**
**States requiring reporting**	**52**	**52**	**52**	**52**	**52**	**N**	**N**	**52**	**52**
**1994**									
**Cases**	**0**	**50**	**8**	**119**	**39**	**N**	**N**	**17**	**96**
**States reporting**	**0**	**11**	**1**	**21**	**14**	**N**	**N**	**5**	**29**
**States requiring reporting**	**52**	**52**	**52**	**52**	**52**	**N**	**N**	**52**	**51**
**1995**									
**Cases**	**0**	**24**	**19**	**98**	**23**	**1**	**0**	**9**	**117**
**States reporting**	**0**	**9**	**4**	**24**	**14**	**1**	**0**	**4**	**24**
**States requiring reporting**	**52**	**52**	**52**	**52**	**52**	**U**	**U**	**51**	**N**
**1996**									
**Cases**	**0**	**25**	**22**	**112**	**4**	**5**	**0**	**5**	**88**
**States reporting**	**0**	**10**	**4**	**29**	**4**	**4**	**0**	**3**	**24**
**States requiring reporting**	**52**	**52**	**51**	**52**	**52**	**U**	**U**	**52**	**N**
**1997**									
**Cases**	**0**	**31**	**22**	**98**	**6**	**14**	**0**	**4**	**101**
**States reporting**	**0**	**8**	**5**	**26**	**5**	**6**	**0**	**3**	**24**
**States requiring reporting**	**50**	**52**	**51**	**51**	**51**	**U**	**U**	**51**	**N**
**1998**									
**Cases**	**0**	**22**	**29**	**79**	**17**	**4**	**0**	**9**	**96**
**States reporting**	**0**	**6**	**2**	**27**	**7**	**4**	**0**	**4**	**22**
**States requiring reporting**	**52**	**52**	**51**	**50**	**52**	**48**	**48**	**50**	**U**
**1999**									
**Cases**	**0**	**23**	**39**	**82**	**6**	**5**	**1**	**9**	**96**
**States reporting**	**0**	**8**	**5**	**18**	**5**	**2**	**1**	**2**	**27**
**States requiring reporting**	**52**	**52**	**52**	**51**	**52**	**48**	**49**	**51**	**U**

**^a^Abbreviations used: N, not nationally notifiable; U, unknown.** **^b^Reports from 50 U.S. states, Washington, D.C., and New York City.** **^c^Includes wound and unspecified botulism.**

We describe disease-specific trends in demographic characteristics and geographic and seasonal distribution of selected conditions caused by critical biologic agents reported to NNDSS. These diseases and conditions include anthrax, botulism, brucellosis, cholera, plague, tularemia, and selected viral encephalitides. By identifying patterns of endemic disease associated with critical agents, we establish a baseline against which future disease incidence can be compared. This process should allow easier identification of unusual reports of disease incidence, which in turn will enhance the ability of the public health community to identify and investigate outbreaks.

## Methods

### Data and Sources

We analyzed NNDSS data voluntarily reported to CDC from state health departments from 1992 to 1999 (*11*). As of 1999, a total of 56 infectious diseases or conditions with public health surveillance case definitions (*12*,*13*) were considered nationally notifiable, as agreed upon by the Council of State and Territorial Epidemiologists and CDC (*14*). Each year, the Council and CDC review the list of nationally notifiable infectious diseases to determine whether conditions should be added or removed as new pathogens emerge or disease incidence changes (*15*). Based on state-specific health priorities, each state independently determines which of the nationally notifiable diseases should be made notifiable (i.e., legally reportable by healthcare providers or laboratories to the public health system within their jurisdiction). As a result, not all nationally notifiable diseases are legally reportable in all states. With some variation by jurisdiction, the completeness of public health surveillance is dependent on healthcare providers and laboratories submitting disease incidence or laboratory reports to local and county health departments, who then forward reports to the state health departments (*16*). Each week, health departments in 50 states, New York City (a separate reporting jurisdiction from New York State), the District of Columbia, and 5 U.S. territories compile surveillance data from their reporting sites and voluntarily transmit disease incidence data to CDC through the National Electronic Telecommunications System for Surveillance.

Conditions associated with critical biologic agents that were nationally notifiable, reported to NNDSS, and included in this study were anthrax, botulism, brucellosis, cholera, plague, tularemia, and selected viral encephalitides. Botulism is reported as two distinct conditions: foodborne botulism and other or unspecified forms of botulism, including wound botulism. All of the study conditions, except tularemia and selected viral encephalitides, were designated as nationally notifiable throughout the study period. Other than tularemia, only cases reported for those diseases designated as nationally notifiable and from states in which the disease was legally reportable were analyzed. Although tularemia was deleted from the nationally notifiable disease list in 1995 because of decreasing incidence, the disease remained reportable in most states, and the annual number of cases reported to NNDSS remained stable in subsequent years; therefore, tularemia incidence data for the entire study period were included in the analysis.

### Analysis

Incidence rates were calculated for the demographic and geographic descriptors of sex, age (grouped as <1 year, 1–4, 5–14, 15–24, 25–39, 40–64, and >65 years), racial category (American Indian or Alaska Native, Asian or Pacific Islander, black, white, and other), Hispanic ethnicity, and state of residence. Seasonal incidence (spring, summer, fall, and winter) was examined on the basis of data reported with one of three types of dates: onset date, date of diagnosis, or date of laboratory result.

Average annual age-, sex-, race-, ethnicity-, and state-specific disease incidence rates for the period 1992–1999 were estimated by averaging the total annual number of case counts by subcategory, and dividing by the study’s mid-year (1995) U.S. population. State-specific annual incidence rates were calculated by using postcensus estimates for July 1, 1992, through July 1, 1998, and population projections for 1999 from the U.S. Bureau of the Census. Incidence rates were calculated per 1 million population because of the small number of cases reported to NNDSS during the study period. Rates were not calculated for extremely rare conditions (anthrax and western equine encephalitis) or for conditions for which data were not collected in all years in the study period (eastern equine encephalitis). Data from U.S. territories were excluded in the analysis.

To provide an example of how historical disease incidence data may be used to assess the likelihood of a reported incident case in the future, we estimated the probability that a given reported case would have the distribution of age, sex, race, ethnicity, geographic residence, and season occurrence using the following formula: P(case) = P(age) x P(sex) x P(race) x P(ethnicity) x P(geographic residence) x P(season). The probability is derived from the NNDSS surveillance data and is calculated under the assumption that these demographic and geographic variables are independent.

## Results

Disease reports for seven conditions caused by critical biologic agents were available for analysis by using NNDSS data for 1992 through 1999 (Table 1). The number of reported cases and incidence rates of the diseases examined in this study, excluding botulism and eastern equine encephalitis, declined or remained stable in the United States during the study period. Tularemia and brucellosis were the most frequently reported diseases (111 and 102 cases/year on average, respectively, yielding the highest estimated incidence rates of 42.1 and 38.7 cases/1 million persons/year, respectively). The least commonly reported diseases were anthrax, with only one case reported in 1992, and western equine encephalitis, with one case reported in 1999.

In general, sex-specific incidence rates were higher among male patients than among female patients for most study diseases. However, rates for foodborne botulism were higher among female than among male patients (Table 2). The age-specific incidence rates varied by disease. Most reported cases of study diseases were in persons >25 years of age; the exceptions were tularemia (highest rates were in children 1–14 years of age) and foodborne botulism (highest rates were in infants <1 year of age).

**Table 2:** Reported cases of conditions caused by critical biologic agents, by demographic characteristics and seasonal occurrence, National Notifiable Disease Surveillance System, United States, 1992–1999^a,b^

Demographic characteristics	Botulism, foodborne		Botulism, other^c^		Brucellosis		Cholera		Plague		Tularemia^d^		
	Cases (%)	Rate^e^	Cases (%)	Rate	Cases (%)	Rate	Cases (%)	Rate	Cases (%)	Rate	Cases (%)	Rate	
**Sex**													
Male	101 (45.3)	9.8	86 (58.1)	8.4	487 (59.9)	47.4	82 (36.8)	8.0	41 (53.2)	4.0	587 (66.3)	57.1	
Female	120 (53.8)	11.2	61 (41.2)	5.7	316 (38.9)	29.4	83 (37.2)	7.7	32 (41.6)	3.0	291 (32.9)	27.1	
Sex not stated	2 (0.9)	NC	1 (0.7)	NC	10 (1.2)	NC	58 (26.0)	NC	4 (5.2)	NC	7 (0.8)	NC	
**Age group (y)**													
<1	21 (9.4)	68.7	3 (2.0)	9.8	8 (1.0)	26.2	1 (0.4)	3.3	0 (0.0)	C	5 (0.6)	16.4	
1–4	1 (0.4)	0.8	2 (1.4)	1.6	34 (4.2)	27.1	7 (3.1)	5.6	3 (3.9)	2.4	100 (11.3)	79.6	
5–14	9 (4.0)	3.0	1 (0.7)	0.3	94 (11.6)	31.0	4 (1.8)	1.3	10 (13.0)	3.3	189 (21.4)	62.3	
15–24	15 (6.7)	5.2	3 (2.0)	1.0	150 (18.5)	51.8	13 (5.8)	4.5	0 (13.0)	3.5	59 (6.7)	20.4	
25–39	45 (20.2)	8.9	59 (39.9)	11.7	231 (28.4)	45.7	40 (17.9)	7.9	17 (22.1)	3.4	128 (14.5)	25.3	
40–64	88 (39.5)	15.2	75 (50.7)	12.9	229 (28.2)	39.5	71 (31.8)	12.2	23 (29.9)	4.0	243 (27.5)	41.9	
>65	36 (16.1)	13.4	4 (2.7)	1.5	58 (7.1)	21.6	30 (13.5)	11.2	13 (16.9)	4.8	141 (15.9)	52.5	
Age not stated	8 (3.6)	NC	1 (0.7)	NC	9 (1.1)	NC	57 (25.6)	NC	1 (1.3)	NC	20 (2.3)	NC	
**Race**													
White	110 (49.3)	6.3	49 (33.1)	2.8	415 (51.0)	23.8	74 (33.2)	4.2	46 (59.7)	2.6	602 (68.0)	34.5	
Black	2 (0.9)	0.8	5 (3.4)	1.9	53 (6.5)	20.0	3 (1.3)	1.1	0 (0)	NC	24 (2.7)	9.1	
American Indian or Alaska Native	72 (32.3)	399.3	0 (0)	NC	1 (0.1)	5.6	0 (0)	NC	23 (29.9)	127.6	89 (10.1)	493.6	
Asian or Pacific Islander	2 (0.9)	2.7	0 (0)	NC	10 (1.2)	13.3	21 (9.4)	28.0	0 (0)	NC	2 (0.2)	2.7	
Other	1 (0.4)	NC	0 (0)	NC	7 (0.9)	NC	3 (1.3)	NC	0 (0)	NC	0 (0)	NC	
Race not stated	36 (16.1)	NC	94 (63.5)	NC	327 (40.2)	NC	122 (54.7)	NC	8 (10.4)	NC	168 (19.0)	NC	
**Ethnicity**													
Hispanic	29 (13.0)	13.3	53 (35.8)	24.3	468 (57.6)	214.5	81 (36.3)	37.1	7 (9.1)	3.2	12 (1.4)	5.5	
Non-Hispanic	113 (50.7)	6.0	66 (44.6)	3.5	143 (17.6)	7.6	56 (25.1)	3.0	62 (80.5)	3.3	407 (46.0)	21.6	
Ethnicity not stated	81 (36.3)	NC	29 (19.6)	NC	202 (24.8)	NC	86 (38.6)	NC	8 (10.4)	NC	466 (52.7)	NC	
Seasonal occurrence**^f,g^**													
Spring	49 (22.0)	x	26 (17.6)	x	220 (27.1)	x	38 (17.0)	x	19 (24.7)	x	244 (27.6)	x	
Summer	33 (14.8)	x	33 (22.2)	x	215 (26.4)	x	37 (16.6)	x	35 (45.5)	x	417 (47.1)	x	
Fall	35 (15.7)	x	48 (32.4)	x	129 (15.9)	x	32 (14.3)	x	12 (15.6)	x	97 (11.0)	x	
Winter	25 (11.2)	x	37 (25.0)	x	142 (17.5)	x	96 (43.0)	x	2 (2.6)	x	52 (5.9)	x	
Eligible date not reported	81 (36.3)	x	4 (2.7)	x	107 (13.2)	x	20 (9.0)	x	9 (11.7)	x	75 (8.5)	x	
**Total**	223 (100)	10.6	148 (100)	7.0	813 (100)	38.7	223 (100)	10.6	77 (100)	3.7	885 (100)	42.1	

^a^Abbreviations used: NC, not calculable; x, rate not calculated. ^b^Reports from 50 U.S. states, Washington D.C., and New York City. ^c^Includes wound and unspecified botulism. ^d^Not nationally notifiable 1995–1998 ^e^Average annual incidence rate. ^f^Includes data reported using one of the following date types only: onset date, date of diagnosis, or date of laboratory result. ^g^Spring includes March, Ap ril, and May; summer includes June, July, and August; fall includes September, October, and November; winter includes December, January, and February.

Race and ethnicity information was incompletely reported in NNDSS. More than 50% of reported cases of unspecified forms of botulism and cholera lacked information regarding race. Disease incidence varied among racial groups. High incidence rates for foodborne botulism, plague, and tularemia were identified in American Indians or Alaska Natives, and the highest incidence rates for cholera and infant botulism were identified in Asian or Pacific Islanders. The average annual disease-specific incidence rates for Hispanic persons were higher than the rates for non-Hispanic persons for most study diseases; the exceptions were plague and tularemia. Tularemia and plague had apparent seasonal patterns: >50% of cases occurred in the summer months (June, July, August). Almost half of reported cholera cases occurred in the winter season (December, January, February) (Figure, Table 2).

**Figure:**
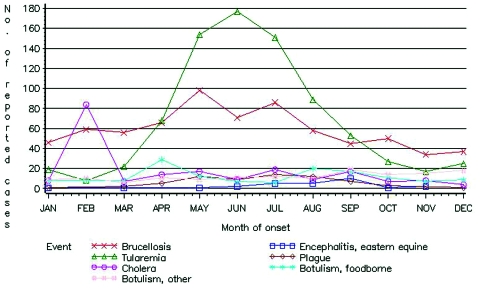
Reported cases of conditions caused by critical biologic agents, by month of onset, National Notifiable Disease Surveillance System, United States, 1992–1999. Cases are reported with one of the following types of dates: onset date, date of diagnosis, or date of laboratory result. Reports are from the 50 U.S. states, Washington, D.C., and New York City.

Table 3 lists the conditions caused by critical biologic agents in rank order by number of reported cases and incidence rates by state of residence; Table 4 gives the geographic region of residence for case-patients. Plague and tularemia incidence demonstrated marked geographic distribution patterns. The highest incidence rates and number of cases of plague (86% of total plague cases) were reported from the mountain region (Montana, Idaho, Wyoming, Colorado, New Mexico, Arizona, Utah, and Nevada); the highest incidence rates and number of cases of tularemia (78% of total tularemia cases) were reported from states in the mountain and the west central regions of the United States. In addition, >60% of botulism case-patients resided in the Pacific region. However, for most other conditions, the states reporting the highest number of cases did not have the highest incidence rates by place of residence. One exception was Alaska, which reported over twice the number of cases and almost 20 times the incidence rate for foodborne botulism compared with the states with the next highest case counts and incidence rates.

**Table 3:** Conditions caused by critical biologic agents, ranking by number of reported cases and incidence rates (per 1 million population) by state of residence, National Notifiable Disease Surveillance System, United States,^a^ 1992–1999

Disease	Rank by reported cases					Rank by incidence rate						
	Rank	State	No. of cases			Rank			State		Average annual incidence rate	
Botulism, foodborne												
	1	Alaska	72			1			Alaska		1,493.7	
	2	Washington	33			2			Washington		75.7	
	3	Texas	27			3			Idaho		75.0	
	4	California	25			4			Wyoming		26.1	
	5	Idaho	7			5			Colorado		20.0	
Botulism, other^b^												
	1	California	128			1			D.C.		51.5	
	2	New Mexico	3			2			California		42.1	
	3	NYC	3			3			New Mexico		25.4	
	4	D.C.	2			4			Mississippi		10.6	
	5	Mississippi	2			5			Utah		7.3	
Brucellosis												
	1	California		215			1		Wyoming		156.5	
	2	Texas		200			2		Texas		133.0	
	3	N. Carolina		58			3		N. Carolina		100.7	
	4	Illinois		53			4		Iowa		92.3	
	5	Florida		31			5		Arizona		87.1	
Cholera												
	1	California		115				1		Nevada	130.4	
	2	Nevada		16				2		California	45.5	
	3	Texas		14				3		Hawaii	42.4	
	4	Louisiana		7				4		Alaska	20.8	
	5	Arizona		6				5		Louisiana	20.2	
Plague												
	1	New Mexico		35				1		New Mexico	258.9	
	2	Arizona		14				2		Arizona	40.7	
	3	Colorado		11				3		Colorado	36.7	
	4	California		9				4		Wyoming	26.1	
	5	Utah		3				5		Utah	19.2	
Tularemia^c^												
	1	Arkansas			211			1		S. Dakota	1,268.0	
	2	Missouri			158			2		Arkansas	1,061.5	
	3	S. Dakota			74			3		Montana	531.4	
	4	Oklahoma			62			4		Missouri	371.3	
	5	Montana			37			5		Oklahoma	236.7	

^a^Reports from 50 U.S. states, Washington, D.C., and New York City (NYC). ^b^Includes wound and unspecified botulism. ^c^Not nationally notifiable 1995–1998.

**Table 4:** Reported cases of conditions caused by critical biologic agents, by geographic region of residence, National Notifiable Disease Surveillance System, United States

	Botulism					
Geographic region^a^	Foodborne	Other	Brucellosis	Cholera	Plague	Tularemia
	Cases (%)	Cases (%)	Cases (%)	Cases (%)	Cases (%)	Cases (%)
New England	1 (0.5)	1 (0.7)	9 (1.1)	7 (3.1)	0 (0.0)	11 (1.2)
Middle Atlantic	9 (4.0)	4 (2.7)	20 (2.5)	16 (7.2)	0 (0.0)	17 (1.9)
East North Central	4 (1.8)	1 (0.7)	82 (10.1)	12 (5.4)	0 (0.0)	46 (5.2)
West North Central	2 (0.9)	1 (0.7)	37 (4.6)	3 (1.4)	0 (0.0)	296 (33.5)
South Atlantic	14 (6.3)	4 (2.7)	116 (14.3)	14 (6.3)	0 (0.0)	32 (3.6)
East South Central	10 (4.5)	2 (1.4)	20 (2.5)	0 (0.0)	0 (0.0)	24 (2.7)
West South Central	28 (12.6)	0 (0.0)	224 (27.6)	21 (9.4)	1 (1.3)	283 (32.0)
Mountain	21 (9.4)	5 (3.4)	65 (8.0)	28 (12.6)	66 (85.7)	114 (12.9)
Pacific	134 (60.1)	130 (87.8)	240 (29.5)	122 (54.7)	10 (13.0)	62 (7.0)
Total	223 (100.0)	148 (100.0)	813 (100.0)	223 (100.0)	77 (100.0)	885 (100.0)

^a^New England includes Maine, New Hampshire, Vermont, Massachusetts, Rhode Island, and Connecticut; Middle Atlantic includes New York, New York City, New Jersey, and Pennsylvania; East North Central includes Ohio, Indiana, Illinois, Michigan, and Wisconsin; West North Central includes Minnesota, Iowa, Missouri, North Dakota, South Dakota, Nebraska, and Kansas; South Atlantic includes Delaware, Maryland, District of Columbia, Virginia, West Virginia, North Carolina, South Carolina, Georgia, and Florida; East South Central includes Kentucky, Tennessee, Alabama, and Mississippi; West South Central includes Arkansas, Louisiana, Oklahoma, and Texas; Mountain includes Montana, Idaho, Wyoming, Colorado, New Mexico, Arizona, Utah, and Nevada; Pacific includes Washington, Oregon, California, Alaska, and Hawaii.

Tables 2–4 present descriptive NNDSS disease incidence data with which to estimate the probability that a reported incident case with selected demographic, geographic, and seasonal characteristics would occur. For example, if the next reported case of brucellosis is in a 30-year-old non-Hispanic white man residing in Florida and occurs in the summer, under the assumption that these studied variables are independent, the probability of occurrence of this case would be 0.02% [P (brucellosis case-patient 1)=P (page 25–39) × P (non-Hispanic) × P (white) × P (male) × P (Florida) × P (summer)=P (28.4%) × P (17.6%) × P (51%) × P (59.9%) × P (3.8%) × P (30.5%) = 0.015%]. Similarly, if the next two reported tularemia case-patients are a 50-year-old non-Hispanic white man in the West South Central United States with onset in the summer (case-patient 1) and a 20-year-old non-Hispanic black woman in the West South Central region with onset in the summer (case-patient 2), then P (tularemia case-patient 1) = 0.86%, and an analogous calculation could be made for the subsequent case, P (tularemia case-patient 2) = 0.004%. Therefore, the probability that those two cases would have the observed characteristics would be P (cases 1 and 2) **=** P (case-patient 1) x P (case-patient 2) = 0.86% x 0.004%= 3.4^–07^.

## Discussion

Early detection of and response to a bioterrorist attack are crucial to decrease illness and deaths, especially in the event of a covert attack with a biologic agent (*17*). To accurately identify unusual or aberrant events prospectively among reports to NNDSS, we characterized the baseline, or endemic, disease incidence. These baseline data can be used by healthcare providers and public health department staff to compare endemic disease distributions and future reported disease incidence in their jurisdictions. From 1992 through 1999, all diseases caused by critical bioterrorist agents occurred at very low incidence rates in the United States. The most common diseases, tularemia and brucellosis, had only approximately 100 cases per year reported to NNDSS. Therefore, each case report of any of these conditions should be considered a sentinel event. Anthrax, eastern equine encephalitis, western equine encephalitis, and plague are so rare that even one case of these diseases should elicit immediate public health investigation and action.

Even with such low incidence, we identified patterns in disease incidence that better prepare us to identify potential bioterrorism events. In this analysis, certain diseases appear to be endemic in certain geographic areas (e.g., foodborne botulism in Alaska, brucellosis and plague in the western states, and tularemia in the central United States). Sporadic disease incidence outside of these regions might indicate aberrant activity. Similarly, certain diseases were common among certain demographic groups. For example, our study indicated a high cholera incidence rate in Asians or Pacific Islanders and a high botulism incidence rate in American Indians and Alaska Natives. Higher incidence rates for brucellosis and tularemia occurred in men and person ∃25 years of age (*18*). Reports of cases clustered in different demographic groups might suggest unusual disease activity potentially associated with bioterrorism or an opportunity for targeted prevention activities.

An explanation of these identified disease incidence patterns becomes clear when we examine disease-specific literature. Since 1989 and before the recent bioterrorism-related anthrax events, only one case of anthrax was reported in the United States, a marked decrease from a yearly average of 130 cases in the early 20th century (*19*–*21*). The decline in human disease caused by the critical agents is believed to have directly resulted from decreased incidence of animal diseases associated with these agents after animal vaccination was implemented. Most outbreaks of foodborne botulism in the United States, especially in Alaska, have been associated with home-prepared foods, including fermented fish (*22*–*25*). High cholera incidence rates in western states and among Asians or Pacific Islanders have previously been associated with travel to cholera-endemic areas of the world (*26*,*27*). The marked seasonal distribution of cholera in the winter season resulted from a large outbreak associated with exposure on a commercial airline flight in February 1992 (*27*). Plague and tularemia are zoonotic diseases with recognized geographic and temporal distributions similar to those of the human cases reported to NNDSS (*28*–*33*). These patterns are probably associated with the distribution of wild rodents or domestic mammal reservoirs and hosts in the western United States or arthropod vector activity in the central states during the summer months.

Given historical trends of studied conditions, disease-specific formulas derived from the surveillance data can be used to estimate the probability that a given series of N cases of the disease would have the distribution of age, race, sex, ethnicity, and seasonal occurrence that was observed. The probability of disease occurrence estimated in this analysis was based on the assumption that these studied variables are independent. In fact, sequentially reported cases would likely cluster temporally. Therefore, the season-specific probability used in the formula to estimate the likelihood of disease cluster may be underestimated. In most cases, the probability derived from these surveillance data gives us the information on expected probability of endemic disease occurrence. Therefore, while further evaluation is needed, this information may be used to compare with current disease incidence data and may serve to set reasonable thresholds for use by health departments considering initiating an epidemiologic investigation of a suspected outbreak or incident case report.

The list of critical biologic agents also includes agents that could be spread through contaminated food or water (e.g., *Salmonella* spp. or *Shigella* spp.). Because diseases caused by these food- and waterborne agents are more common in the United States compared to bioterrorism-associated diseases such as plague or tularemia, outbreaks associated with these more common agents will most likely continue to be identified through ongoing surveillance and health communication efforts that require a strong public health infrastructure. With the increasing availability of electronic health outcome data, CDC and certain states are evaluating the application of statistical aberration detection algorithms to state and national notifiable disease incidence data to aid the rapid identification of unusual disease incidence patterns (*34*). To support early detection of potential bioterrorist events, these or similar methods have also been applied at the state and local public health system level, where data are more timely (compared with national NNDSS data).

Even at the local and state level, however, passive notifiable disease reporting from healthcare providers and laboratories is often not timely or complete (*35*,*36*). Disease incidence reported in this analysis is likely an underestimate because of underreporting by physicians and healthcare providers. The recent terrorism-associated anthrax attacks highlighted the need for healthcare provider recognition of the syndromes associated with potential bioterrorist agents and rapid communication of relevant health outcome information between the healthcare community and the public health system. Physician case reporting is generally more complete for conditions that cause severe clinical illnesses (e.g., plague) but less complete for diseases that cause mild clinical illness (*37*). In the United States, the completeness of notifiable disease reporting has been estimated to range from 9% to 99% (*37*–*40*). Healthcare providers lack awareness of reporting requirements, and changes in surveillance case definitions may also lead to underreporting of notifiable diseases (*37*). In addition, state- and disease-specific differences in surveillance practices or in the amount of resources applied to surveillance efforts affect how actively cases are solicited or identified. Increasing awareness among healthcare providers and laboratories regarding accurate and rapid identification of conditions related to critical agents and local reporting requirements and methods is necessary to establish and maintain communication between the medical and public health communities. Increased resources (both human and technical) for surveillance at the state and local level may augment disease reporting as well.

Although most diseases caused by critical biologic agents are nationally notifiable conditions, diseases have historically been added to or deleted from the nationally notifiable disease list on the basis of criteria that did not include their etiologic agent’s potential use in a bioterrorist event. Therefore, not all conditions caused by critical biologic agents are nationally notifiable diseases. For example, tularemia was temporarily removed from the nationally notifiable disease list in 1995 because of decreasing incidence. Eradicated diseases (e.g., smallpox [*41**–**43*]) are not technically nationally notifiable, nor are emerging infections (e.g., Nipah virus infection and the viral hemorrhagic fevers). However, local and state public health code typically supports the reporting of unusual events that pose a public health threat. Even when nationally notifiable, however, not all conditions caused by critical biologic agents are designated as reportable in all states because states determine which conditions should be reportable in their state based on their own public health priorities and needs. Among the diseases examined in this study, only foodborne botulism was reportable in all states for the entire study period. To enhance and expand surveillance for potential bioterrorist events, CDC and the Council of State and Territorial Epidemiologists have recently added Q fever and reinstated tularemia to the list of nationally notifiable diseases. CDC continues to collaborate with the Council of State and Territorial Epidemiologists and state health departments to ensure that all nationally notifiable diseases caused by critical biologic agents are reportable in all states.

Caution should be exercised in interpreting specific incidence rates. Incidence rates for study diseases may also be underestimated because they were calculated on the basis of the U.S. population of all 50 states for the mid-study year of 1995, not limited to the population of reporting states for each year. Although rates might be underestimated, the patterns identified would not likely be affected. Although CDC and the Council of State and Territorial Epidemiologists have defined the standard case definitions for all nationally notifiable diseases, differences exist regarding how states interpret and apply these criteria. For example, although observed incidence rates of foodborne botulism were very high among children aged <1 year, these cases might be infant botulism reported as foodborne botulism. Therefore, standardized application of surveillance case definitions needs to be encouraged. Race and ethnicity information is incomplete in NNDSS data, potentially leading to underestimation of race- and ethnicity-specific incidence rates (*44*,*45*).

Overall, the incidence of conditions caused by critical microbiologic agents with the potential for use in acts of terrorism is low in the United States, as reported to NNDSS. Therefore, each case report should initially be considered a sentinel event requiring further investigation, especially reports from nonendemic regions of conditions with identified geographic distribution patterns. For potential bioterrorism-related conditions that are endemic and have low incidence, nontraditional surveillance methods (e.g., sentinel emergency department surveillance [*46*]) and complementary data sources (e.g., electronic laboratory reporting [*47*]) might be used to complement traditional sources of surveillance data (e.g., NNDSS) and can enhance our ability to detect changes in disease incidence.
